# Lactate programs PBX1 lactylation and mesangial proliferation in lupus nephritis

**DOI:** 10.1172/jci.insight.190838

**Published:** 2025-04-29

**Authors:** Enzhuo Liu, Chenghua Weng, Chenchu Yan, Xingchen Zhu, Xinyue Li, Mengdi Liu, Zhenke Wen, Zhichun Liu

**Affiliations:** 1 Department of Rheumatology, The Second Affiliated Hospital of Soochow University, Soochow University, Suzhou, China.; 2 The Fourth Affiliated Hospital of Soochow University, Institutes of Biology and Medical Sciences, Suzhou Medical College of Soochow University, and; 3 Jiangsu Key Laboratory of Infection and Immunity, MOE Key Laboratory of Geriatric Diseases and Immunology, Soochow University, Suzhou, China.

**Keywords:** Autoimmunity, Metabolism, Lupus

## Abstract

Lupus nephritis (LN) constitutes the most common organ-threatening manifestation of systemic lupus erythematosus (SLE), with the pathological proliferation of mesangial cells (MCs) recognized as a critical factor in its pathogenesis and progression. Self-DNA-containing immune complex (DNA-IC) represents a prime pathogenic factor in SLE, yet its pathological effect on MCs remains unclear. In the present study, we elucidated the mechanism underlying the excessive proliferation of MCs following the recognition of DNA-IC in patients with LN. Here, we pinpointed that the excessive proliferation of MCs was attributed to an anomalous transition from the G_1_ to the S phase of the cell cycle in patients with LN. Mechanically, the dysfunction of P27 protein resulted in the aberrant G_1_-S phase transition, and the phenomenon was closely related to the ubiquitin-mediated degradation of its key transcription factor, PBX1. This degradation was regulated by lactylation of PBX1 in the site of Lys40 residue. The elevated lactylation level of PBX1 protein was caused by the upregulation of glycolysis levels induced by DNA-IC. Accordingly, targeting lactate production in MCs from patients with LN effectively alleviated their renal inflammation and fibrosis progression. Elevated lactate resulted in PBX1 lactylation, leading to excessive proliferation of MCs and, thus, serving as a promising therapeutic target for LN.

## Abstract 2

Immune complexes instruct PBX1 lactylation for excessive proliferation of mesangial cells in human lupus nephritis.

## Graphical abstract

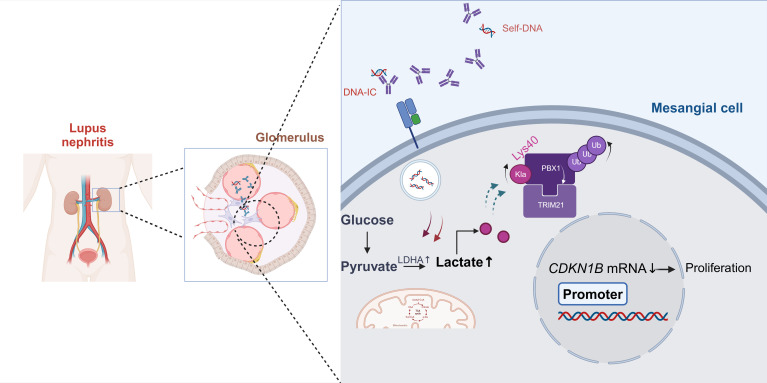

## Introduction

Systemic lupus erythematosus (SLE) is a canonical chronic autoimmune disorder that predominantly affects young women (1). Renal biopsies show that almost all patients with SLE have renal involvement; lupus nephritis (LN) continues to be a main cause of morbidity and death among patients diagnosed with SLE, due to its propensity to progress to end-stage renal disease and limited therapeutic efficacy in refractory cases (2). Glucocorticoid medications, despite being the cornerstone of current therapeutic strategies, are frequently associated with an increased risk of infections and a spectrum of adverse effects (3, 4). Meanwhile, newer targeted therapies, including rituximab and belimumab, have demonstrated limited efficacy in reducing mortality rates or preventing the progression to end-stage renal disease (5, 6). The above suggests that treatment of patients with LN may be more focused on the localized immune status and pathological damage in the cells of tissues.

Patients with SLE often have a deficiency of DnaseI (7), resulting in high levels of circulating self-DNA. Self-DNA is a key trigger in SLE, driving the production of anti-double-stranded DNA (anti-dsDNA) antibodies and the formation of self-DNA-containing immune complex (DNA-IC) (8, 9). The role of DNA-IC in SLE pathogenesis is well established; however, its impact on specific tissues and the mechanisms driving diverse cytopathological effects remain unclear. Extensive research has shown that self-DNA is recognized by intracellular DNA sensors (8, 10–12). Investigating the impact of DNA-IC on kidney tissue and identifying potential DNA sensors is crucial for understanding the pathogenesis of SLE.

The glomerulus, characterized by its unique capillary collateral structure and high-pressure environment, serves as the primary site of DNA-IC deposition and the principal target tissue in LN (13). Mesangial cells (MCs), constituting 30%–40% of renal cells, are glomerular stromal cells essential for the formation of glomerular capillary collaterals and the regulation of glomerular filtration pressure (14, 15). Furthermore, MCs exhibit a range of immunological functions (16, 17). The mesangial region is the main deposition area of DNA-IC and the earliest site of pathological damage and manifestation (12, 18). MCs are the primary responders to deposited DNA-IC. Upon external injury, they initiate glomerular repair and adaptation through proliferation and extracellular matrix production (19, 20). Additionally, MCs tend to show excessive proliferation and stromal fibrosis when the injury persists (2). MC proliferation is a hallmark of LN progression, with mesangial proliferative glomerulonephritis being the glomerular injury pattern most prone to advancing toward renal interstitial fibrosis (21). Current studies indicate that multiple factors induce proliferation of MCs in patients with LN (17, 22, 23), but the role of DNA-IC is unclear. Studies have shown that a multitude of DNA sensors are expressed in MCs (24–26), with the potential to be activated by DNA and thereby exert influence on the disease process. However, the precise effects of DNA-IC on MCs in the renal tissue of patients with LN and the mechanisms of their recognition remain largely unknown.

This study aimed to investigate the mechanisms underlying MC proliferation in patients with LN. Our findings revealed an increase in glycolysis in MCs induced by DNA-IC from patients with LN. Additionally, we identified PBX1 lactylation as a key molecular factor driving excessive MC proliferation in LN.

## Results

### DNA-IC induces the proliferation of MCs through promoting G_1_-S phase transition.

**Figure 1:**
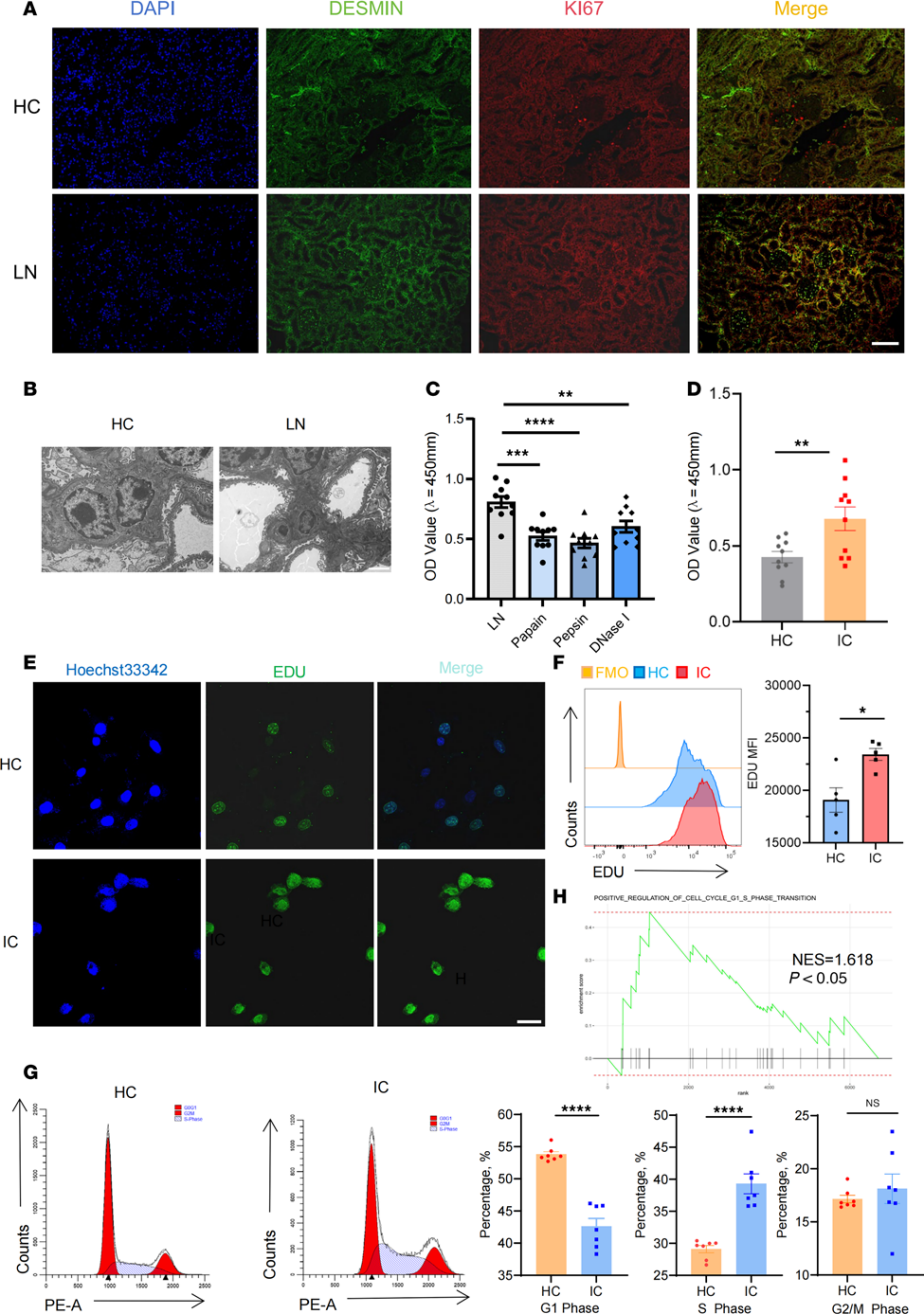
DNA-IC promotes excessive proliferation of MCs. (**A** and **B**) Humanized chimeras were constructed by infusing NSG mice intraperitoneally with PBMCs (1 × 10^7^cells/per mouse) isolated from patients with SLE or healthy donors. A total of 4 chimeras were included in each experimental group. (**A**) The proliferation of MCs was demonstrated by dual-color immunostaining of DESIN (green) and KI67 (red). The nuclei were marked with DAPI, which produced a blue fluorescence. (**B**) Transmission electron microscopy was employed to observe the glomerular ultrastructure of humanized chimeras. (**C**) MCs were conditioned with SLE plasma that had been pretreated with pepsin (pepsin/IgG = 1:60) or papain (papain/IgG = 1:60) and DnaseI (50 μL working buffer for 5 μg DNA) in order to eliminate IgG and DNA component (papain cleaves the hinge region of IgG into Fab/Fc fragments, while pepsin degrades Fc regions). The proliferation of MCs was detected by the CCK-8 assay. MCs were stimulated with DNA-IC (80 IU/mL) isolated from plasma of patients with SLE or IgG from healthy donors for 1 day. MCs were stimulated with DNA-IC isolated from plasma of patients with SLE (IC group) or healthy donors (HC group). (**D**) Effect of DNA-IC on proliferative activity of MCs detected by CCK-8 assay (*n* = 10). (**E** and **F**) EdU assay revealed that the percentage of EdU^+^ cells was significantly higher in LN MCs. (**G**) Cell cycle detection was analyzed in LN MCs and HC MCs by the cell cycle assay kit (*n* = 7 for each group). (**H**) Gene set enrichment analysis of single-cell data from SYD997, enriched in genes involved in cell_cycle_G1_S_phase_transition progression. NES, normalized enrichment score. Lines with whiskers show the mean ± SEM. Scale bar: 100 μm (**A**), 50 μm (**E**), 2 μm (**B**). *P < 0.05, **P < 0.01, ***P < 0.001, ****P < 0.0001 with ANOVA plus Turkey’s method (**C**) and paired *t* test (**D**, **F**, and **G**).

To investigate the pathological function of MCs in patients with LN, we conducted an enrichment analysis of the differentially expressed genes and found a high tendency for extracellular matrix production and inflammation response in MCs from patients with LN (Supplemental Figure 1A; supplemental material available online with this article; https://doi.org/10.1172/jci.insight.190838DS1), indicating a significant correlation between MCs and the development of renal fibrosis and inflammation in patients with LN. To verify the damage of MCs in patients with LN and their relationship with disease prognosis, immune-deficient NSG mice were immune reconstituted with PBMCs from patients with SLE (Supplemental Figure 1B). Such humanized LN chimeras exhibited a robust levels of human anti-dsDNA IgG antibodies, urine protein, and renal deposition of human IgG antibodies (Supplemental Figure 1, C and D), accompanied by pathological renal manifestations associated with MC lesions: glomerular mesangial expansion, inflammatory cell infiltration, basement membrane involvement, and increased collagen fiber deposition (Supplemental Figure 1, E–G). Immunofluorescence was conducted to ascertain the expression of DESMIN and KI67, revealing robust proliferation of MCs in humanized LN chimeras (Figure 1A). Electron microscopy showed tangible electron density deposition in humanized LN chimeras, suggesting that immune complex may be a contributing factor in this range of outcomes (Figure 1B). These findings demonstrated that MCs display a notable proliferation trend in human LN.

To clarify the role of DNA-IC in the proliferation of MCs, we stimulated MCs with plasma from patients with SLE and healthy donors. Remarkably, the plasma from patients with SLE could effectively induce MC proliferation, and this phenomenon was not due to a decrease in apoptosis (Supplemental Figure 1, H–L). We pretreated SLE plasma with papain or pepsin to remove IgG and with DnaseI to degrade self-DNA and subsequently tested MC proliferation. Both treatments resulted in a reduction of excessive proliferation (Figure 1C). Thus, we isolated DNA-IC from plasma of patients with SLE (LN MCs) and IgG from healthy donors (HC MCs) and obtained their direct effect on MC proliferation, and subsequent evaluation yielded consistent results (Supplemental Figure 1, M and N, and Figure 1, D–F), suggesting that DNA-IC from plasma of patients with SLE is the key that induces MC proliferation. The process of cell cycle transition represents the intrinsic molecular mechanism underlying cell proliferation. Subsequently, a cell cycle analysis was conducted to explore the intrinsic mechanism of cell proliferation influenced by DNA-IC. Cell cycle analysis revealed that DNA-IC–induced proliferation in LN MCs was associated with a disrupted G_1_-S phase transition (Figure 1G), and this phenomenon of cell cycle abnormality in MCs was also corroborated by the results of single-cell analysis of patients with LN (Figure 1H). Collectively, DNA-IC drove excessive MC proliferation and cell cycle transition in LN.

### Deficiency of PBX1 protein causes the aberrant G_1_-S phase transition of MCs.

**Figure 2:**
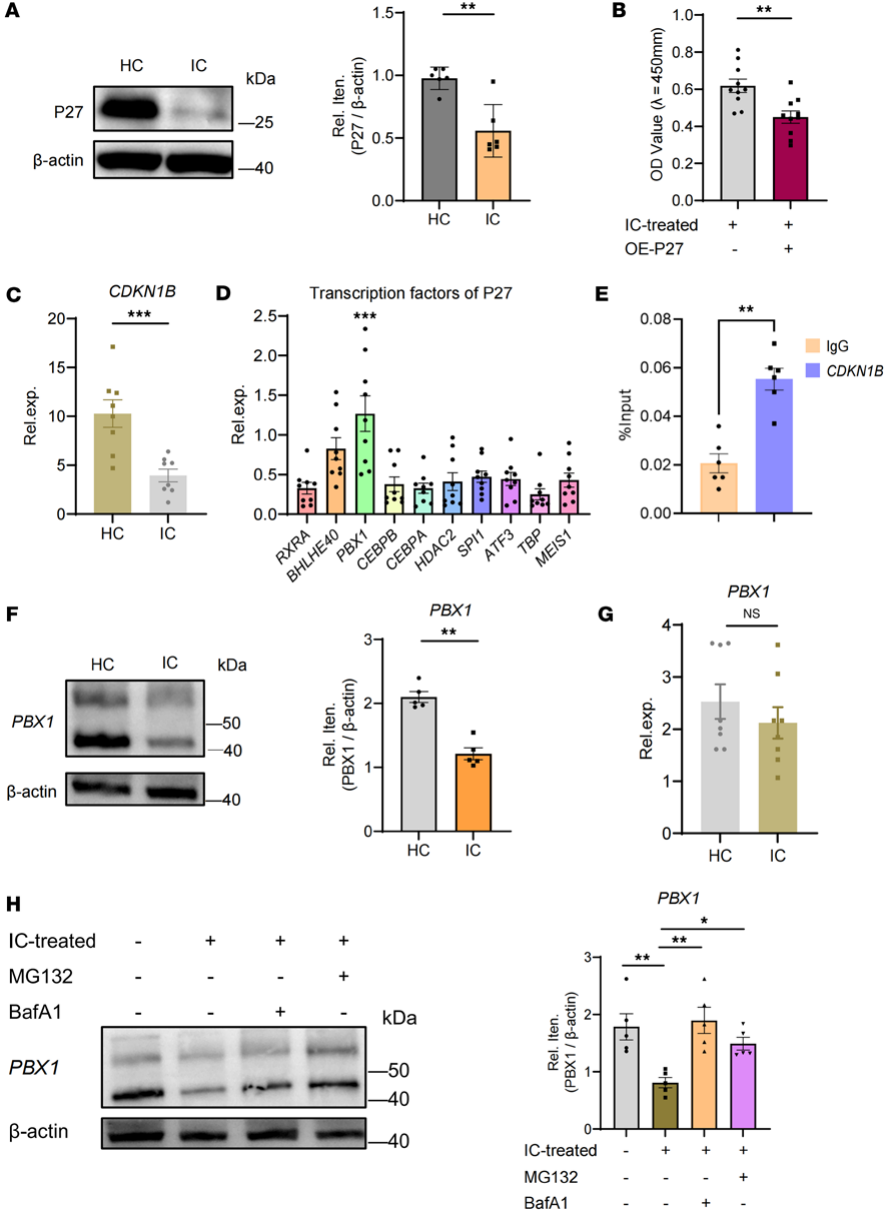
Deficiency of PBX1 drives MC proliferation. (**A**) Immunoblotting of P27 proteins in LN and HC MCs. β-Actin from same gel is shown under the corresponding blots as loading control (*n* = 6). (**B**) The proliferation of LN MCs was rescued upon P27 overexpression (OE), as reflected by CCK-8 assay (*n* = 10). (**C**) RT-qPCR detected the expression of *CDKN1B* mRNA of LN MCs (*n* = 8). (**D**) Database screening to identify the upstream transcription factors of P27. RT-qPCR analysis of the upstream transcription factors of P27 in HC MCs (*n* = 9 for each group). (**E**) The occupancy of PBX1 on the *CDKN1B* promoter was determined by CHIP assay (*n* = 6). (**F**) Immunoblot analysis of PBX1 in LN and HC MCs. β-Actin from same gel is shown under the corresponding blots as loading control (*n* = 5). (**G**) RT-qPCR detected the expression of *PBX1* mRNA in HC and LN MCs (*n* = 8). (**H**) The PBX1 protein level was tested by immunoblot analysis in the presence or absence of the proteasome inhibitor MG132 (2 μM) or lysosome inhibitor bafilomycin A1 (0.1 μM) for 4 hours in LN MCs. The same samples were run on a separate gel for detecting β-actin (*n* = 5). Lines with whiskers show the mean ± SEM. *P < 0.05, **P < 0.01, ***P < 0.001 with ANOVA plus Turkey’s method (**D** and **H**) and paired *t* test (**A**–**C** and **E**–**G**).

GSEA analysis was conducted to confirm the differentially expressed genes enriched in the cell cycle–related pathway in MCs from patients with LN (Supplemental Figure 2A). Specifically, *CDKN1B* (the gene that encodes the P27 protein) exhibited the most prominent change (Supplemental Figure 2B), indicating its unique function in the excessive proliferation of MCs. Furthermore, DNA-IC from patients with LN could induce the downregulation of P27 protein in MCs (Figure 2A). To confirm the role of P27 in promoting DNA-IC–induced MC proliferation, we overexpressed P27 in LN MCs, which resulted in a reduction of proliferation (Supplemental Figure 2C and Figure 2B). Concurrently, the expression of *CDKN1B* mRNA exhibited a pattern consistent with its protein expression (Figure 2C), indicating a salient downregulation of P27 transcription in LN MCs. Using the Signaling Pathways Project (http://www.signalingpathways.org/index.jsf), we identified the 10 transcription factors with the strongest binding affinity score for the promoter region for *CDKN1B* and selected PBX1 for further investigation due to its high expression levels (Figure 2D). Furthermore, to determine the binding capacity of PBX1 to the promoter for *CDKN1B* in MCs, a ChIP–quantitative PCR (ChIP-qPCR) assay was conducted (Figure 2E and Supplemental Figure 2D). Meanwhile, to assess the effect of PBX1 on P27 levels, Flag-PBX1–overexpressed plasmid was transfected into HEK293 cells (Supplemental Figure 2E). After a 48-hour incubation period, examination revealed a remarkable increase in P27 protein levels (Supplemental Figure 2F), which led to the conclusion that *CDKN1B* promoter occupancy by PBX1 and PBX1 acts as a positive regulator of P27. Further investigation revealed a decrease in PBX1 protein level, while mRNA expression remained unchanged in LN MCs (Figure 2, F and G). The aforementioned results indicated that the deficiency of PBX1 occurred at the protein level. Two principal mechanisms of protein degradation in the cell have been identified: the ubiquitin-proteasome system and the autophagy progress. Therefore, we employed proteasome inhibitor MG132 and bafilomycin A1 (BafA1) to inhibit the ubiquitin-proteasome and lysosomal pathways, respectively, and observed that these agents effectively restored PBX1 protein level (Figure 2H). The PBX1-P27 axis may account for the abnormal G_1_-S phase transition in LN MCs.

### TRIM21-mediated K29- and K63-linked ubiquitination drives PBX1 deficiency.

**Figure 3:**
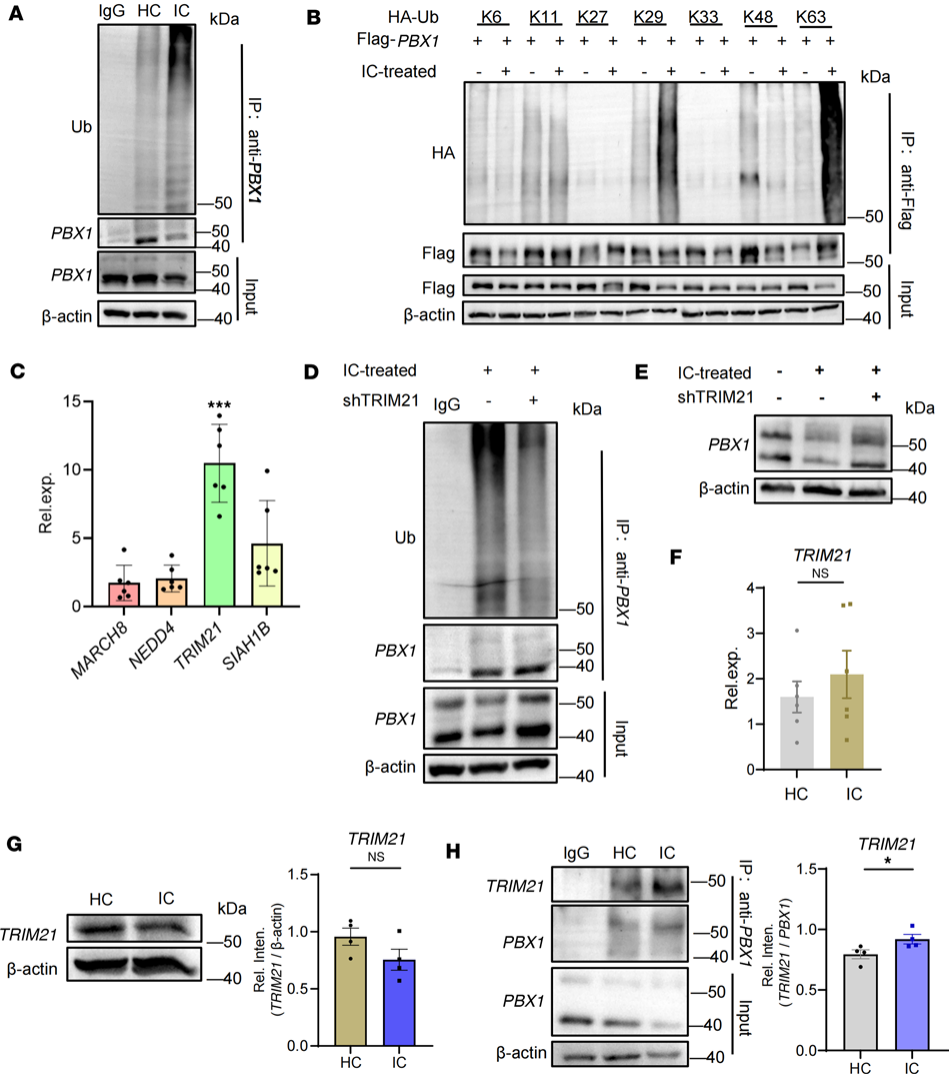
Ubiquitination of PBX1 accounts for its deficiency in MCs. (**A**) The endogenous immunoprecipitated PBX1 protein from LN and HC MCs was detected in order to analyze the level of ubiquitination. IP: PBX1 were analyzed on a different gel using the same biological samples; input: PBX1 and β-actin were detected on a separate gel from the same samples (*n* = 7). (**B**) HEK293 cells were transfected with different types of HA-Ub plasmids and stimulated with the presence or absence of DNA-IC. Flag-PBX1 was immunoprecipitated for detection of ubiquitination. IP: Flag was analyzed on a different gel using the same biological samples; input: Flag and β-actin were detected on a separate gel from the same samples. (**C**) Mass spectrometry was conducted to screen for possible E3 ligases that bind with PBX1 in LN MCs. E3 ubiquitin ligases were screened from both the HC and LN groups, and only proteins with more than 1 peptide are shown. Furthermore, RT-qPCR was used to analyze the relative expression of ligase mRNA, which was compared in order to define candidate E3 ubiquitin ligase. Data are from 6 independent experiments. (**D** and **E**) TRIM21 shRNAs were transfected into MCs, and immunoblotting was used to detect PBX1 protein level and the ubiquitination level of endogenous immunoprecipitated PBX1 protein (in **D**, lanes were run from the same sample on different gels using immunoblot and immunoprecipitation; in **E**, lanes were run on the same gel). (**F**) *TRIM21* mRNA expression was compared between LN and HC MCs using RT-qPCR in 6 independent experiments. (**G**) Immunoblot analysis of TRIM21 protein in LN and HC MCs. Lanes were run on the same gel (*n* = 4). (**H**) The binding of PBX1 to TRIM21 in LN and HC MCs was detected by coimmunoprecipitation. Lanes were run from the same sample from different gel using immunoblot and immunoprecipitation. Lines with whiskers show the mean ± SEM. *P < 0.05, ***P < 0.001 with ANOVA plus Turkey’s method (**C**) and paired *t* test (**A** and **F**–**H**).

To explore the mechanisms underlying the PBX1 deficiency, we tested the ubiquitination of PBX1, revealing an increased PBX1 ubiquitination in LN MCs, which was further clarified with prominent elevation at the K29- and K63-linked residues (Figure 3, A and B, and Supplemental Figure 3A). To identify the potential E3 ubiquitin ligases responsible for PBX1 ubiquitination, mass spectrometry was performed to detect PBX1-binding proteins in MCs. The candidates identified were MARCH8, NEDD4, TRIM21, and SIAH1B (Supplemental Figure 3B). We further examined the mRNA expression of these candidates in LN MCs and found that *TRIM21* was notably abundant, suggesting its role in mediating PBX1 ubiquitination and degradation (Figure 3C).

To determine the role of TRIM21 in PBX1 degradation, we selectively silenced TRIM21 in MCs (Supplemental Figure 3C). The results demonstrated that TRIM21 plays a crucial role in mediating PBX1 ubiquitination and degradation (Figure 3, D and E). However, TRIM21 mRNA expression and protein levels were comparable in MCs (Figure 3, F and G). Building upon this result, we hypothesized that the binding capacity between TRIM21 and PBX1 would be aberrant. Therefore, coimmunoprecipitation revealed a notable increase in TRIM21 binding to PBX1 in LN MCs (Figure 3H). Thus, TRIM21-mediated PBX1 ubiquitination leads to PBX1 deficiency in LN MCs.

### DNA-IC enhances glycolysis and lactate generation to promote PBX1 deficiency.

**Figure 4:**
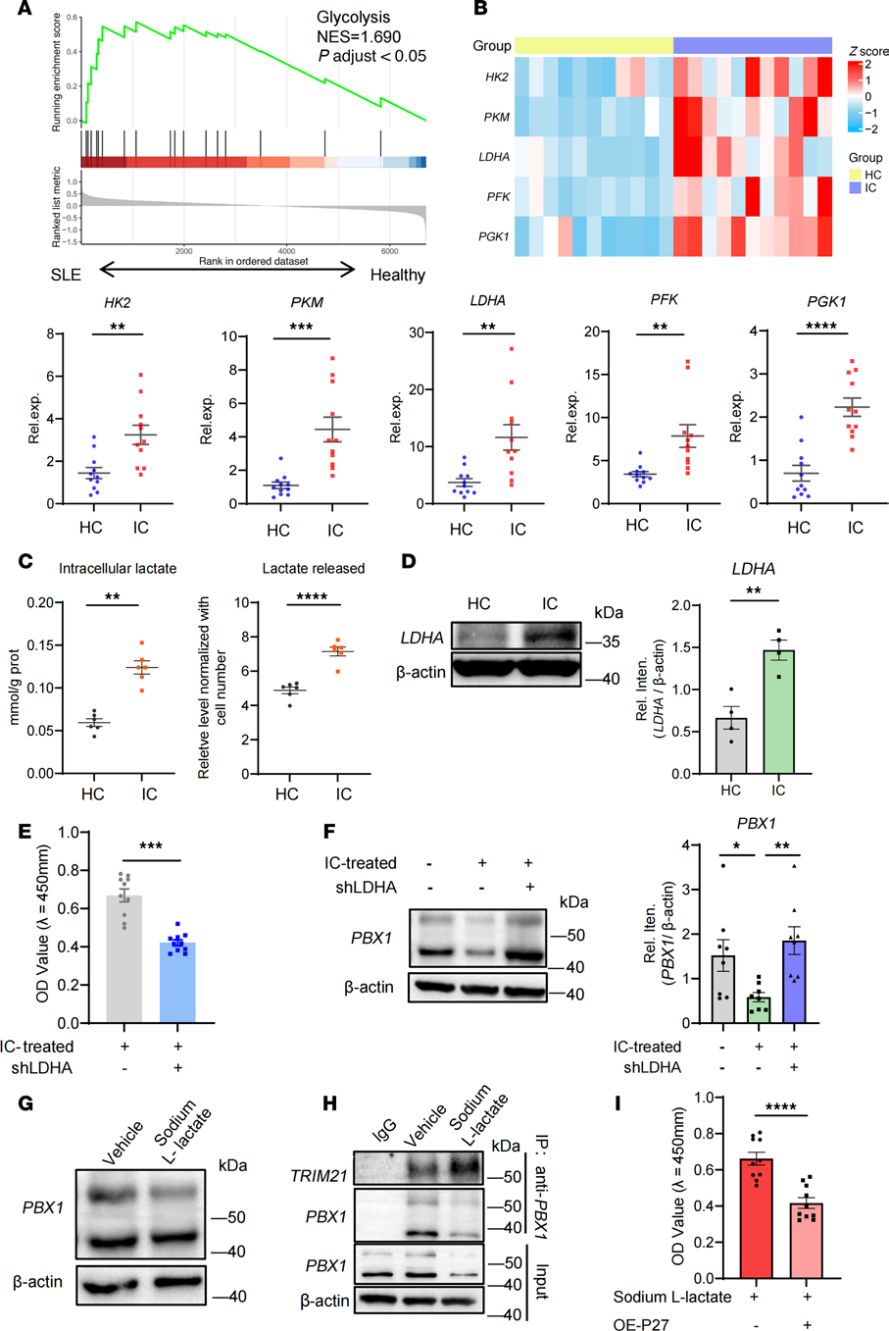
Lactate promotes PBX1 ubiquitination in MCs. (**A**) Gene set enrichment analysis of single-cell data from SDY997, showing that MCs in patients with LN were enriched in genes involved in glycolysis progress. NES, normalized enrichment score. (**B**) mRNA expression of differential genes that regulate key catalytic enzymes involved in the glycolysis progress was compared between LN and HC MCs by RT-qPCR (*n* = 11 for each group). (**C**) Detection of lactate production levels in intracellular lactate content and extracellular lactate release in both LN and HC MCs (*n* = 6 for each group). (**D**) Representative Western blot analysis of LDHA protein level in LN and HC MCs (*n* = 4). (**E** and **F**) LN MCs were transfected with LDHA shRNA. Cell proliferation was detected using the CCK-8 assay, and protein level of PBX1 was determined using immunoblots. (**G**–**I**) MCs were stimulated with sodium L-lactate for 1 day. (**G** and **H**) MCs were stimulated in the presence or absence of sodium L-lactate (30 mM) and tested for protein level of PBX1 and its combination capacity with TRIM21. (**I**) The proliferation induced by sodium L-lactate was rescued upon P27 overexpression, as reflected by the CCK-8 assay. Lines with whiskers show the mean ± SEM. *P < 0.05, **P < 0.01, ***P < 0.001, ****P < 0.0001 with ANOVA plus Turkey’s method (**F**) and paired *t* test (**B**–**E** and **I**). In all cases, for a given condition, representative Western blot images are from the same sample, although the target proteins may have been probed on separate membranes to facilitate clear visualization.

Cell metabolism undergoes alterations in response to various pathological conditions and plays a key role in regulating cellular functions. It was unclear whether LN MCs underwent a characteristic metabolic reprogramming. Subsequent analysis of single-cell data from patients with LN revealed a striking upregulation of the glycolytic pathway (Figure 4A). We observed a upregulation of genes associated with the glycolytic pathway in LN MCs compared with HC MCs (Figure 4B). The level of lactate was substantially upregulated in both intracellular and extracellular of LN MCs (Figure 4C). Meanwhile, the protein level of LDHA was increased (Figure 4D). Thus, the proliferation of LN MCs was accompanied by robust glycolytic metabolism.

To test the potential role of lactate in DNA-IC–induced MC proliferation, MCs were transfected with shLDHA (short hairpin RNA targeting LDHA) to interrupt lactate production (Supplemental Figure 4A); results showed that MC proliferation induced by DNA-IC was alleviated (Figure 4E). Meanwhile, the expression of PBX1 protein was rescued in LN MCs transfected by shLDHA (Figure 4F). Exogenous lactate could promote the proliferation of MCs (Supplemental Figure 4B and Figure 4G). Of note, the binding capacity between PBX1 and TRIM21 was enhanced by exogenous lactate (Figure 4H). The deficiency of PBX1 protein caused by exogenous lactate could be reinforced by P27 overexpression (Figure 4I).

The aforementioned findings indicated that lactate plays a role in the aberrant proliferation of MCs in patients with LN. To further investigate the relationship between DNA-IC and elevated glycolysis in MCs, we used RU.521, H151, E6446, and MCC950 to inhibit the function of some common DNA sensors, including cGAS/STING, TLR9, and NLRP3. The results demonstrated that inhibiting the cGAS/STING pathway effectively reduced lactate production (Supplemental Figure 4C). Altogether, sodium L-lactate acted as a pivotal metabolite through regulation of PBX1 deficiency in LN MCs.

### Lactylation of PBX1 at the Lys40 site facilities its ubiquitination in MCs.

**Figure 5:**
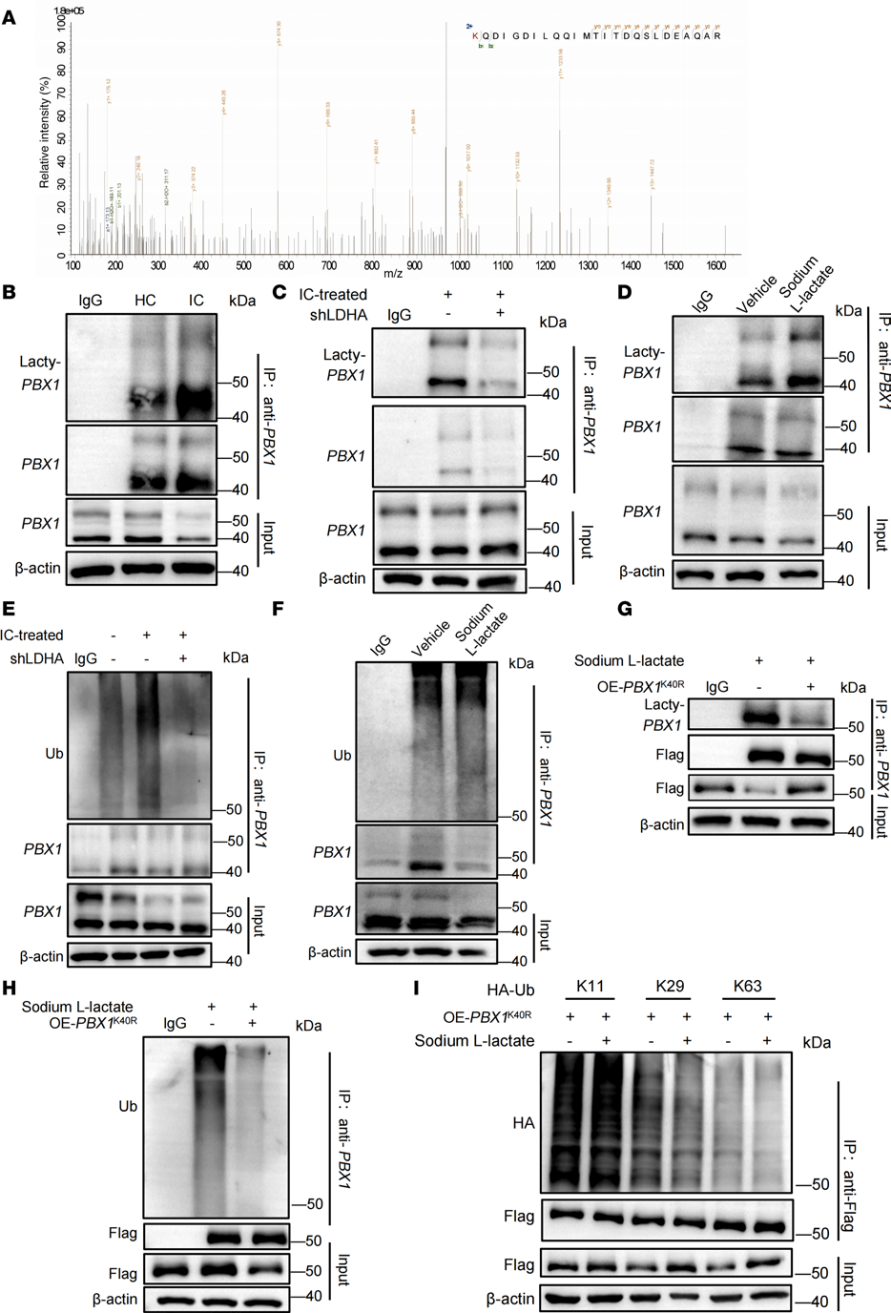
PBX1 lactylation controls its ubiquitination in MCs. (**A**) HEK293 cells were transfected with Flag-PBX1 stimulated with sodium L-lactate for mass spectrometry detection. (**B**) The lactylation level of endogenous immunoprecipitated PBX1 protein was detected in LN and HC MCs. (**C**) Lactylation level of PBX1 in LN and HC MCs that were transfected with LDHA shRNA. (**D**) MCs were stimulated with sodium L-lactate, and lactylation of endogenous immunoprecipitated PBX1 protein was detected using immunoblots. (**E**) LN MCs were transfected or not with LDHA shRNA and tested for the ubiquitination level of endogenous immunoprecipitated PBX1 protein. (**F**) MCs were stimulated in the presence or absence of sodium L-lactate, and the ubiquitination level of endogenous immunoprecipitated PBX1 protein was detected by immunoblots. (**G**–**I**) HEK293 cells were transfected with Flag-PBX1^K40R^ and different types of HA-Ub plasmids and were stimulated with sodium L-lactate. Flag-PBX1 was immunoprecipitated for detecting lactylation and ubiquitination level. In all cases, for a given condition, representative Western blot images are from the same sample, although the target proteins may have been probed on separate membranes to facilitate clear visualization.

To elucidate the possible role of lactate in posttranslational modification (PTM) of PBX1 protein, Flag-PBX1–overexpressed plasmid was transfected into HEK293 cells, followed by treatment with sodium L-lactate and subsequent pull down for mass spectrometry analysis. We identified a lactylation site at the Lys40 residue in PBX1 (Figure 5A). We assessed the lactylation level of PBX1 and found it to be markedly elevated in LN MCs (Figure 5B). Transfection of shLDHA into LN MCs to block intracellular lactate production led to a decrease in PBX1 lactylation (Figure 5C). Conversely, exogenous lactate increased PBX1 lactylation in MCs (Figure 5D).

To explore the relationship between PBX1 ubiquitination and lactylation, we assessed the ubiquitination level of PBX1 in LN MCs transfected with shLDHA and observed a reduction in ubiquitination (Figure 5E). In contrast, exogenous lactate increased PBX1 ubiquitination (Figure 5F), indicating a potential correlation between PBX1 lactylation and ubiquitination.

Since Lys40 residue was the lactylation site of PBX1, a point mutation plasmid was conducted and transfected to HEK293 cells with sodium L-lactate treatment. Subsequent results showed a notable weakened lactylation and ubiquitination of PBX1 (Figure 5, G and H). The mutation of the PBX1 Lys40 residue resulted in a reduction in K29- and K63-linked ubiquitination (Figure 5I). These findings indicated that lactate causes elevated K29- and K63-linked ubiquitination through the lactylation of PBX1 in Lys40 residue.

### Targeting lactate generation alleviates LN progression in humanized chimeras.

Humanized LN chimeras were constructed by intraperitoneal injection of PBMCs derived from patients with LN. We employed the use of LDHA inhibitors (sodium oxamate), which were administered 1 week following the modeling of humanized LN chimeras (27).

**Figure 6:**
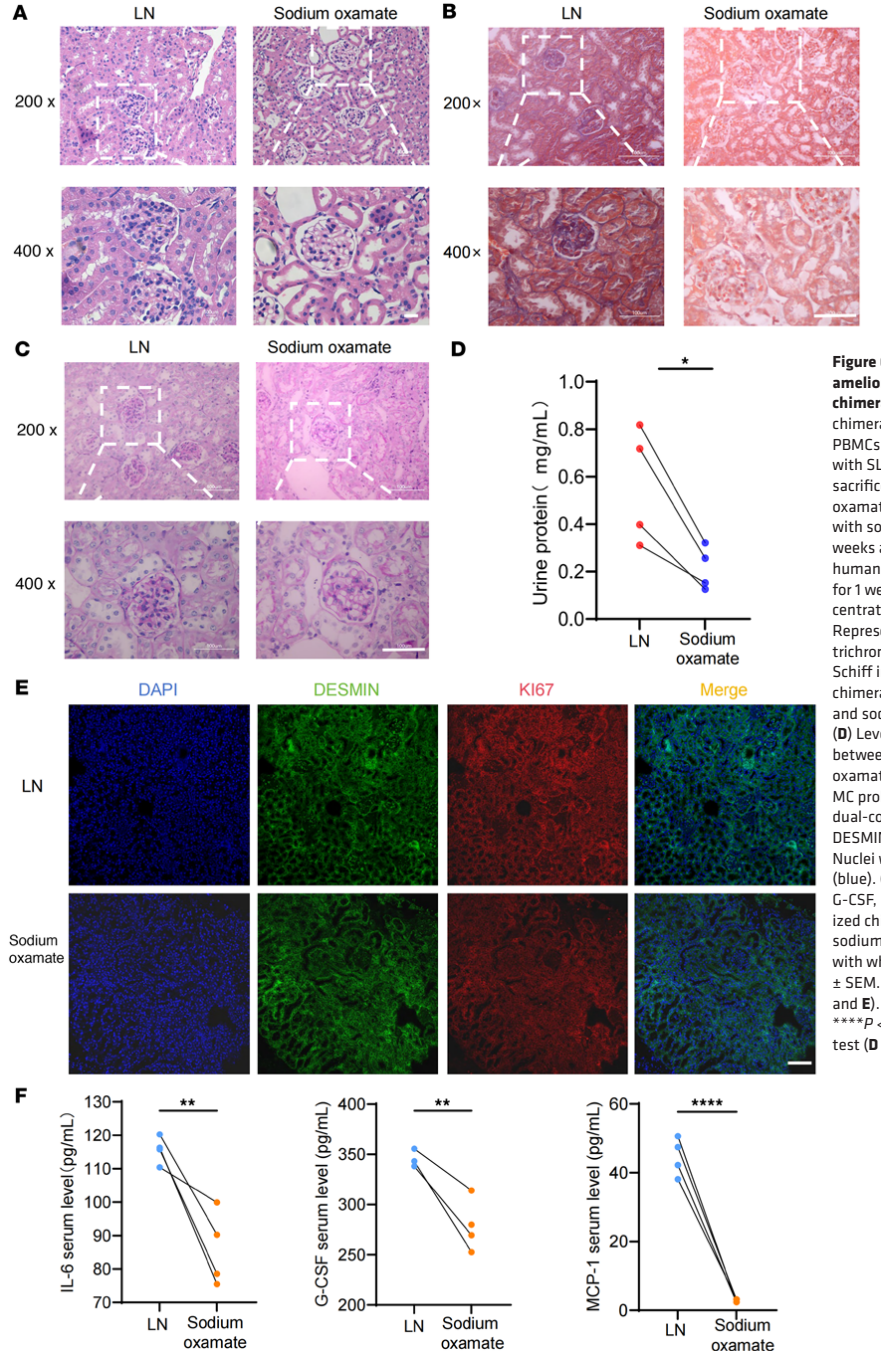
Targeting lactate ameliorates LN in humanized chimeras. Humanized LN chimeras were injected with 10^7^ PBMCs isolated from patients with SLE for 1 month before sacrifice. Those in the sodium oxamate group were injected with sodium oxamate for 3 weeks after experiments with humanized LN chimeras began for 1 week, with a blood concentration of 600 mg/kg. (**A**–**C**) Representative H&E, Masson trichrome, and periodic acid Schiff images in humanized chimera kidneys from the LN and sodium oxamate groups. (**D**) Level of urine protein between the LN and sodium oxamate groups (*n* = 4). (**E**) MC proliferation was shown by dual-color immunostaining of DESMIN (green) and KI67 (red). Nuclei were marked with DAPI (blue). (**F**) Serum level of IL-6, G-CSF, and MCP-1 in humanized chimeras of the LN and sodium oxamate groups. Lines with whiskers show the mean ± SEM. Scale bar: 100 μm (**A**–**C** and **E**). *P < 0.05, **P < 0.01, ****P < 0.0001 with unpaired *t* test (**D** and **F**).

The introduction of sodium oxamate into humanized LN chimeras resulted in a substantial decrease in glomerular inflammatory cell infiltration, renal fibrosis, and glomerular basement membrane destruction (Figure 6, A–C, and Supplemental Figure 5, A and B). Meanwhile, there was an improvement in proteinuria following the inhibition of lactate production (Figure 6D). To corroborate the effect of sodium oxamate on MCs, immunofluorescence staining was employed to depict the proliferation of MCs in humanized chimera kidneys. The result indicated that targeting lactate production could effectively alleviate the excessive proliferation of MCs in patients with LN (Figure 6E). Furthermore, a notable decrease in pro-inflammatory factors of associated with the pathogenesis of LN was obtained (Figure 6F). These findings suggested that the inhibition of lactate production represented a key for the improvement of renal prognosis in human LN.

## Discussion

In patients with LN, MC proliferation contributes substantially to renal fibrosis and inflammation. We identified that this aberrant proliferation results from cell cycle checkpoint dysregulation due to metabolic reprogramming. Mechanistically, lactate-driven PBX1 ubiquitination degradation via Lys40 lactylation affects its occupancy in the *CDKN1B* promoter, highlighting a potential therapeutic target in LN MCs.

The proliferation of MCs serves as a vital indicator in the diagnosis of patients with LN (2, 28), concerning the renal damage and prognosis (2). MCs are fundamental to the formation of glomerular structure (15), maintaining the basic function of glomerulus under physiological conditions. In addition to their structural role, MCs play a key role in local immunomodulation through mechanisms such as immune complex phagocytosis, antigen presentation, and inflammation (17, 29).

The sensing of self-DNA is a critical factor in the pathogenesis of inflammation, cancer and autoimmune diseases (11, 30–32). Previous studies revealed that DNA-IC could induce the activation and proliferation of autoreactive B cells through BCR and TLR9 (33). Self-DNA has been identified as a key trigger of innate activation, capable of inducing anti-dsDNA autoantibodies and forming DNA-IC, thereby sustaining the production of these autoantibodies in patients with SLE (34). Nevertheless, the pathological mechanism of DNA-IC in the local renal environment remains largely unknown. Therefore, an understanding of the manner in which cellular mechanisms within the kidney regulate pathogenic responses to DNA-IC is of significant clinical importance. In current study, we demonstrate that DNA-IC is effective in promoting the aberrant proliferation of MCs in patients with LN.

In recent years, as a transcription factor, PBX1 has been abundantly demonstrated to play a key role in tumorigenesis as well as in the pathogenesis of autoimmune diseases (e.g. SLE), and it is increasingly likely to be a therapeutic target. PBX1 exhibits a “double-edged sword” property in tumors, exhibiting either oncogenic or tumor-suppressive roles, depending on the tissue type and molecular context (35). In recent years, it has been found that a variety of immune cells in patients with SLE exhibit aberrant PBX1 expression, which in turn drives disease progression by affecting their survival and differentiation and contributing to inflammatory amplification. For example, patients with LN have a specific PBX1 deficiency in B cells, resulting in increased B cell survival and plasma cell differentiation, leading to increased antibodies production (36). In addition, PBX1 is also overexpressed in Tregs of patients with LN, which inhibits their numbers and activity, leading to enhanced inflammatory responses (37). A similar phenomenon was found in our study, that a low expression of PBX1 was responsible for the aberrant proliferation of MCs; this may further suggest the importance of PBX1 in the pathogenesis of LN.

The distinction between oxidative phosphorylation and glycolysis as the predominant metabolic pathway for glucose is a defining characteristic of quiescent versus proliferating cells (38, 39). This alteration in the metabolic pattern of rapidly proliferating cells may be attributed primarily to the avoidance of oxygen utilization during the process of cell division, which serves to prevent damage to the newly duplicated DNA (40). Our study demonstrated that the aberrant proliferation of MCs in patients with LN was accompanied by a distinctive elevation in glycolysis. Of interest, lactate, the product of glycolysis, plays a pivotal role in the development and progression of a multitude of diseases (41). In acute inflammatory diseases, lactate functions as a signaling molecule that prompts monocyte differentiation to M2 macrophages (42). In chronic inflammatory diseases, lactate inhibits T cells migration, thereby perpetuating inflammation and inducing IL-17 production by CD4^+^ T cells (43). While the metabolic profile of stromal cells in kidneys from patients with LN has been less extensively investigated, we elucidated that glycolysis represents a distinctive metabolic process occurring in MCs, serving as a key for the pathological alterations of MCs.

Given that protein synthesis is an energy-consuming and slow process, rapid and dynamic responses can be modified through PTMs that alter protein function while restoring homeostasis in vivo (44). The involvement of protein PTMs in disease processes has been substantiated (45). For instance, an imbalance in the ubiquitin-proteasome system has been linked to the development of autoimmune diseases (46), while acetylation has been shown to influence the progression of atherosclerosis by regulating histones and nuclear proteins (47). Lactylation is a recently identified PTM of lysine residues (48). Histone lactylation has been identified as a potential mediator of immune escape in lung cancer (49). The extant research demonstrates that lactylation is not only prevalent in the histone proteome but also in the broader human proteome (50). Herein, we found that elevated lactate levels in MCs of patients with LN facilitated the lactylation of PBX1 protein. In vivo, various PTM processes are not isolated occurrences; rather, they are frequently interconnected and collectively implicated in the regulation of protein homeostasis and function (51). We demonstrated the interaction between the lactylation and ubiquitination of the PBX1 protein.

In summary, we elucidated the mechanisms underlying DNA-IC–induced MC proliferation in patients with LN. Specifically, LN MCs exhibited metabolic reprogramming with elevated glycolysis, resulting in increased lactate production. Lactate acted as a substrate for PBX1 lys40 lactylation, enhancing PBX1 binding to the E3 ubiquitin ligase TRIM21. This interaction led to K29- and K63-linked ubiquitination and subsequent PBX1 degradation, reducing the transcription of the cell cycle inhibitor P27, and thus, promoting abnormal MC proliferation. These findings offer insights into the pathogenesis of LN and potential therapeutic strategies for LN management.

### Limitations of study.

In this study, we focused on the regulatory role of the P27 upstream transcription factor PBX1 and its interaction with lactate metabolism, omitting a comprehensive assessment of other upstream or downstream alternative pathways that might synergistically contribute to the observed phenotypes. Additionally, DNA-IC in patients with SLE has been identified as a crucial driver of MCs, and our study offers preliminary evidence suggesting that it may enhance glycolysis through the cGAS/STING pathway. However, the precise mechanism through which this occurs remains to be elucidated. Furthermore, the humanized LN chimeras used in our study may closely resemble the in vivo environment. However, its phenotype may underestimate the therapeutic potential, and thus, an aggressive nephritis phenotype disease model is essential in transformation research.

## Methods

### Sex as a biological variable.

Since lupus disproportionately affects women, 91% of participants included in this study were female. As for the mouse model, sex was not considered as a biological variable, and only female mice were used in this study.

### Patients and healthy donors.

Patients with SLE were recruited for this study according to the 2019 American College of Rheumatology revised criteria. The new-onset SLE cohort comprised (a) patients who were diagnosed with SLE within the 6 months preceding enrollment and had not undergone treatment and (b) patients with hydroxychloroquine and low-dose corticosteroid (≤10 mg/d) treatment. In contrast, the established SLE cohort included patients with a confirmed SLE diagnosis who had received a range of therapeutic interventions, including major immunosuppressive therapies. Healthy volunteers without concomitant autoimmune disease and renal involvement matched by age, sex, and race were also recruited as a control population. The information regarding all populations is summarized in Supplemental Table 1.

### Cell isolation and culture.

Human glomerular MCs and human embryonic kidney 293 (HEK293) cells were purchased from the Cell Institute of the Chinese Academy of Sciences, Shanghai, China, and were cultured in DMEM high-glucose medium (Gibco) with 100 U/mL penicillin, 0.1 mg/mL streptomycin, and 10% fetal bovine serum (PAN) at 37°C with 5% CO_2_. PBMCs were isolated from the blood of patients with SLE and healthy volunteers using lymphocyte isolation solution according to previous experimental methods (52).

### Humanized LN chimeras.

Human lupus PBMC-NSG chimeras were generated as previously described (53). All mice (Biocytogen Technologies) were housed under specific pathogen–free conditions. For the construction of animal models, 6-week-old female NSG mice were immune reconstituted by intravenous injection of PBMCs (1 × 10^7^ cells/mouse) isolated from patients with LN or healthy donors. One week later, the LDHA inhibitor (oxamic acid sodium, MCE, 600 mg/kg) was injected into the tail vein at a frequency of once a day to inhibit lactate production, and 1 month later, the mice were sacrificed for further experiments.

### Processing of single-cell data.

The single-cell RNA-sequencing dataset (SDY997; https://www.immport.org/home), comprising 24 patients with LN and 10 samples from healthy individuals, was obtained from the ImmPort database. Following quality control, a total of 2,882 single cells and 23,372 genes were retained for subsequent analysis. The kidney cells were clustered using Seurat (v4.2.0) in a stepwise manner. The initial step involved low-resolution clustering, whereby all cells were analyzed collectively, followed by the delineation of a cluster of MCs. In each instance, clustering was conducted subsequent to principal component analysis, based on context-specific variable genes that were identified independently for each set of analyzed cells. The gene sets were then ordered in descending order according to the number of changes in gene expression.

### Transfections and reagents.

The PBX1 overexpression plasmid, P27 overexpression plasmid, LDHA shRNA, and TRIM21 shRNA were obtained from MIAOLING BIOLOGY. Two million cells were transiently transfected with 2 μg of human plasmid expression vectors or empty vectors. We used the following products: Proteasome inhibitor MG-132 (MCE, HY-13259), Bafilomycin A1 (MCE, HY-100558), Sodium L-lactate (Sigma-Aldrich, 867-56-1), the Lactic Acid assay kit (NJJCBIO, A019-2-1), the Annexin V-PE/7-AAD Apoptosis Detection Kit (Yeasen, 40310ES50), the Mouse MCP-1 ELISA Kit (Abmart, AB-B30406A), the Mouse G-CSF ELISA Kit (AB-B22933A), and the Mouse IL-6 ELISA KIT (Abmart, AB-B30998A). All reagents were used in accordance with the manufacturer’s instructions.

### Real-time PCR.

Total RNA was extracted using Trizol (Takara). cDNA was synthesized using HiScript II Q RT SuperMix for qPCR (Vazyme) with reverse transcription (RT-qPCR) (Vazyme). qPCR analyses were conducted using SYBR Green qPCR Master Mix (Bimake), and gene expression was normalized to 18S. The primers are listed in the Supplemental Table 2.

### Immunoblotting and immunoprecipitation.

Immunoblotting and immunoprecipitation assays were performed as standard protocols. Primary antibodies were used as followed: PBX1 (Proteintech, 18204-1-AP, 1:2,500), P27 (Proteintech, 25614-1-AP, 1:3,000), Ubiquitin (Proteintech, 10201-2-AP, 1:1,500), LDH-A (Santa Cruz, sc-137243, 1:1,000), L-Lactyl Lysine (PTMBIO, PTM-1401, 1:1,000), TRIM21 (Proteintech, 67136-1-lg, 1:2,000), β-actin (Proteintech, 66009-1-lg, 1:12,000), Flag Tag (ZENBIO, 390002, 1: 1,000), and HA-Tag (ZENBIO, 390001, 1:1,000).

### Immunofluorescence.

Immunofluorescence was performed to visualize the expression of intracellular proteins. Frozen sections of kidney tissue were incubated with Ki67 anti-rabbit mAb (Abcam, ab16667, 1:100) and Desmin anti-mouse mAb (Santa Cruz, sc-65983, 1:100) overnight at 4°C. Next, sections were incubated with Dylight 549 Goat Anti-Rabbit IgG (Abbkine, A23320, 1:200) and DyLight 488, Goat Anti-Mouse IgG (Abbkine, A23210, 1:200) for 1 hour at room temperature. Nuclei were stained with DAPI. Images were observed using the confocal (Nikon).

### CCK-8 assay.

Cells were seeded in 96-well plates at 1 × 10^4^ cells/well, After 24 hours of planting, a Cell Counting Kit-8 (CCK-8) assay (Beyotime, C0038) was conducted, and the relative numbers of live cells was evaluated using 10% CCK-8 diluted in standard culture media at 37°C for 30 minutes. Quantification was performed by measuring the OD at 450 nm.

### DNA-IC isolation and identification.

DNA-IC was isolated from plasma of patients with SLE while IgG was isolated from plasma of healthy donors using the PurKine antibody purified kit (protein A/G) (Abbkine, KTP2070). The presence of DNA was confirmed on a 1% agarose gel, while the presence of IgG antibodies was confirmed by SDS-PAGE.

### Treatment of plasma with nucleases and proteases.

The plasma was treated with DnaseI for 10 minutes or incubated with papain and pepsin for 30 minutes and 2 hours at 37°C, respectively.

### Cell cycle assay.

Human glomerular MCs in the exponential growth phase were plated at a density of 2 × 10^5^ cells per well in 6-well plates. Prior to the commencement of the experiments, the cell cycle was normalized by subjecting the cells to a serum-free medium for a period of 24 hours. Subsequently, the cells were harvested and subjected to staining using the Cell Cycle Assay Kit — PI/RNase Staining (DOJINDO). The data were analyzed using the ModFit software.

### EdU stain analysis.

The population of DNA-replicating cells was assessed using a BeyoClick EdU Cell Proliferation Kit with Alexa Fluor 488 (Beyotime). Images were visualized with a laser confocal microscope (Nikon Eclipse Ti). The EdU^+^ rate was calculated as the ratio of the number of EdU^+^ cells to the number of Hoechst-stained cells.

### CUT&RUN assay.

The ChIP-qPCR assay was performed using the Hyperactive pG-MNase CUT&RUN Assay Kit (Vazyme, HD101-01). The PBX1 antibody (Proteintech, 18204-1-AP, 1:2,500) was used for ChIP. Specific DNA fragments were obtained, purified and the integrity of the DNA was confirmed by gel electrophoresis. The recovered DNA was then subjected to PCR/qPCR amplification using primers that targeted specific genomic regions of *CDKN1B*. The primer sequences utilized are as follows: F 5′-AGACTCGCCGTGTCAATCAT-3, R 5′-TGTCCTCAGAGTTAGCCGGA-3′.

### Statistics.

All data are presented as mean ± SEM. Paired and unpaired 2-tailed Student’s *t* tests were used for 2-group comparisons as appropriate. One-way ANOVA with Tukey’s method was used for comparison of more than two groups. The significance level was set to 0.05. Data were analyzed using the GraphPad prism (version 9.0).

### Study approval.

Written informed consent was obtained from all human participants involved in the study, and all experiments involving human samples were conducted in accordance with the Declaration of Helsinki. Experiments with NSG mice were performed in accordance with the ARRIVE guidelines. All experiments were approved by the Ethics Committee of Soochow University.

### Data availability.

All relevant data supporting the findings of this study were included in the manuscript or its supplemental material. Data are available in Supporting Data Values file and can be obtained from the corresponding authors upon reasonable request.

## Author contributions

ZW and ZL designed and supervised the study. EL, CW, XZ, CY, XL, and ML were responsible for the collection of samples, the performance of experiments, and the analysis of data. The manuscript was prepared by ZW and EL with input from all authors.

## Supplementary Material

Supplemental data

Supporting data values

## Version 1 04/29/2025

In-Press Preview

## Version 2 06/09/2025

Electronic publication
