# Supplementary material for: Lactate programs PBX1 lactylation and mesangial proliferation in lupus nephritis
Source: JCI Insight. 2025 Apr 29;10(11):e190838. doi: 10.1172/jci.insight.190838 (PMC12220973; doi:10.1172/jci.insight.190838)
Supplement: Supplemental data [file jciinsight-10-190838-s208.pdf]

Supplementary Figure 1

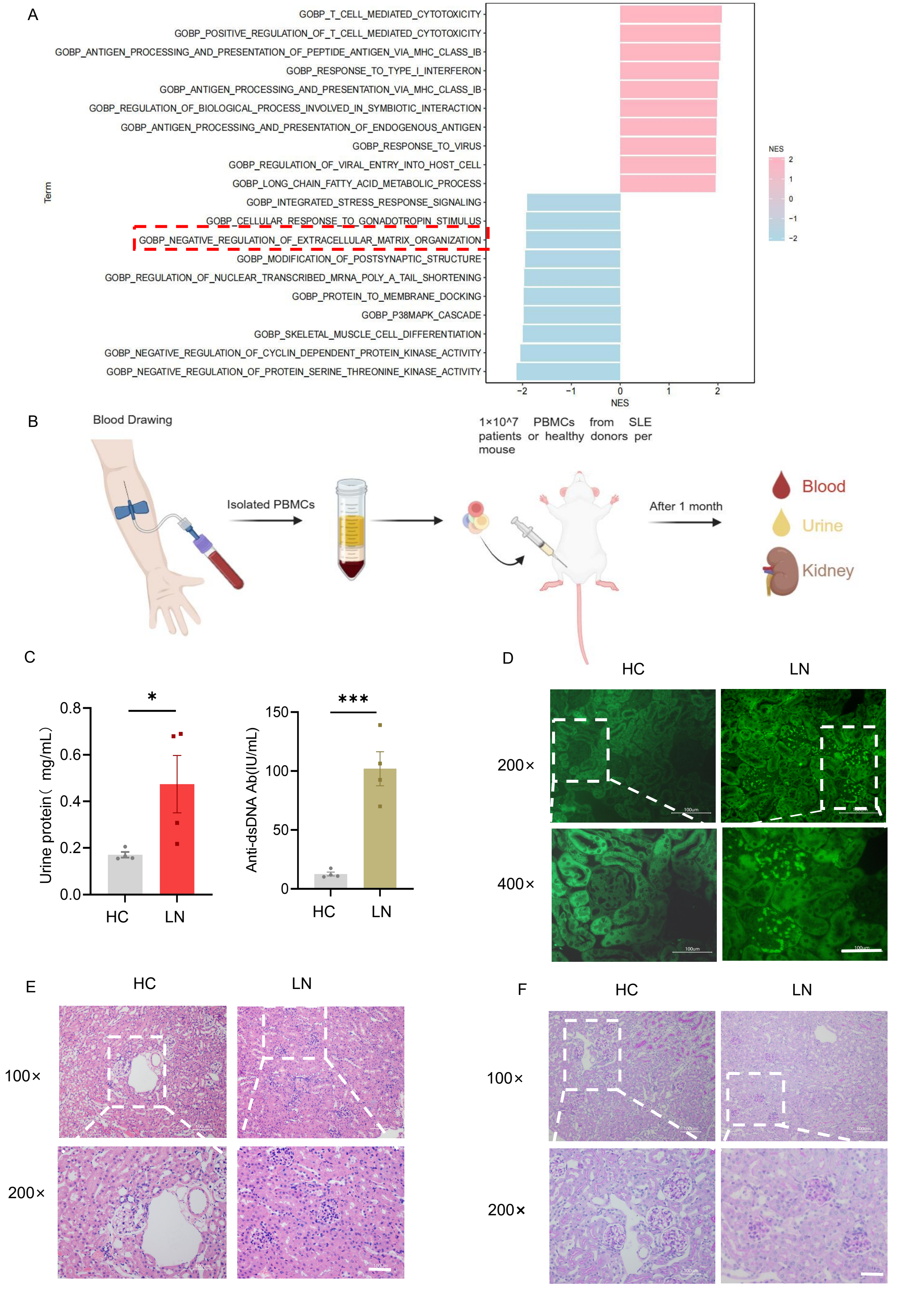

Supplementary Figure 1

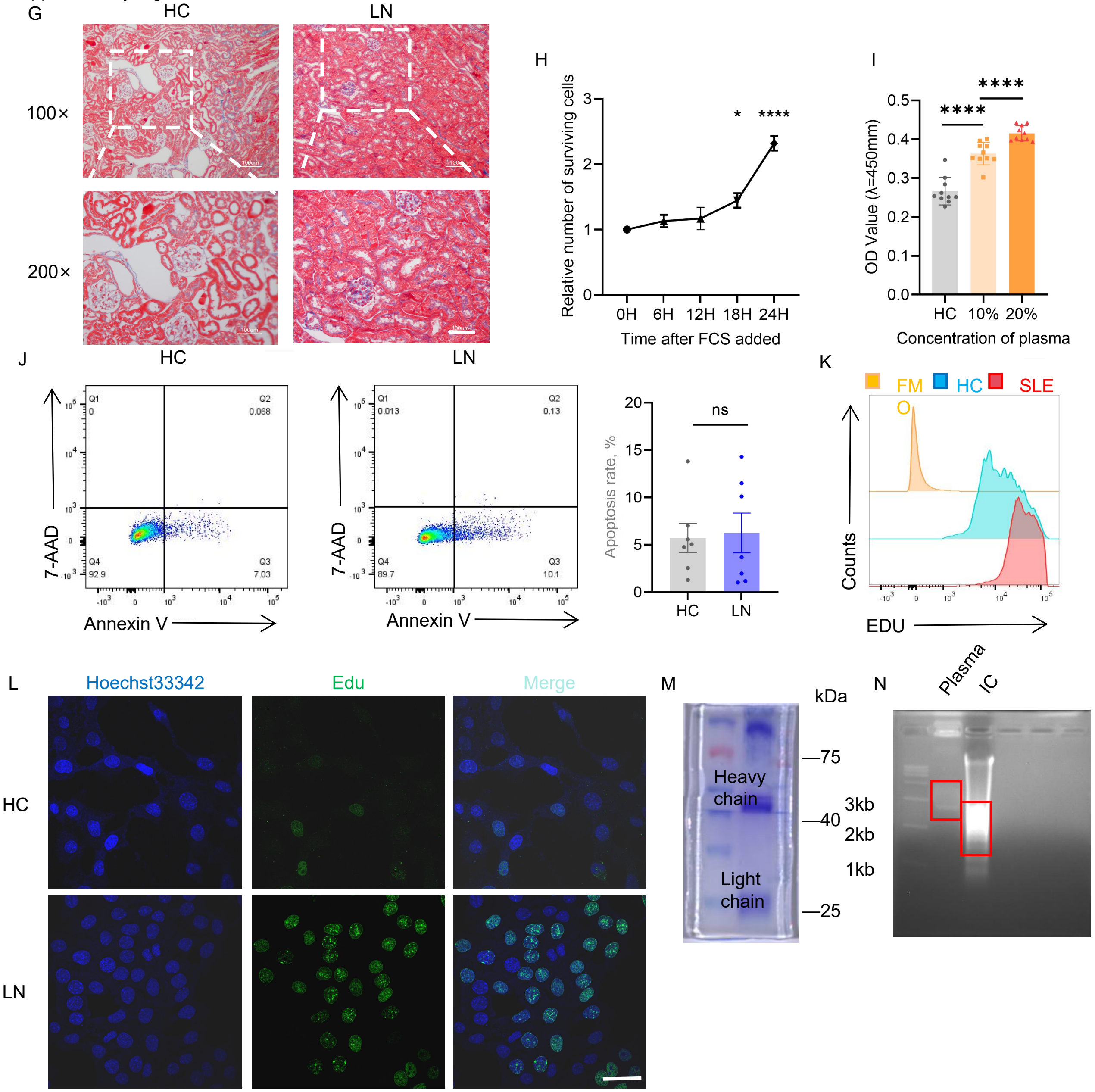

Fig.S1 Mesangial cells proliferation and LN kidney damage

(A) Single-cell data from published profiling data (SDY997) of healthy control and LN patients' kidneys was analyzed to determine the top 20 terms enriched from differentially expressed genes in MCs with adjusted P values less than 0.05. (B) Experimental design for construction of humanized chimeras. (C-G) 1×10^{7} PBMCs isolated from SLE patients or healthy donors and subsequently injected intraperitoneally into NSG mice to establish humanized HC and LN chimeras. (C) Urine protein level (left), level of anti-dsDNA antibodies (right). (D) Renal deposition of human IgG antibodies. (E) H&E staining. (F) PAS staining. (G) Masson staining. (H) To detect MCs division cycle, MCs were cultured with complete medium (CM) after fetal calf serum free for 24 hours, and relative cells number were tested by CCK-8 kit (n = 10, each group). (I) MCs were stimulated with different concentration of plasma from SLE patients, plasma from healthy donors as control, CCK-8 kit was used for detecting relative cells number (n = 10). (J) The apoptosis rate of MCs were detected by Annexin V apoptosis analysis kit (n=7). (K, L) EdU analysis was performed to detect the DNA replication rate, EDU+ MCs were revealed by EDU cell proliferation kit through flow cytometry and Immunofluorescence experiments. (M) Electrophoresis was performed to detect the presence of IgG from DNA-IC isolated from patients with LN. (N) Plasma nucleic acid from SLE patients was extracted using a method that involved the use of phenol-chloroform. IC-associated nucleic acid was isolated from the same group. The extracted nucleic acid was then subjected to visualization through ethidium bromide staining of a 1.0% agarose gel. lines with whiskers show the mean±SEM. Scale bar =100μm (D-G), 50μm (L) *p < 0.05, ***p < 0.001, ****p < 0.0001 with ANOVA plus Turkey's method (H-I), and unpaired t-test (C). PAS, periodic acid Schiff.

Supplementary Figure 2

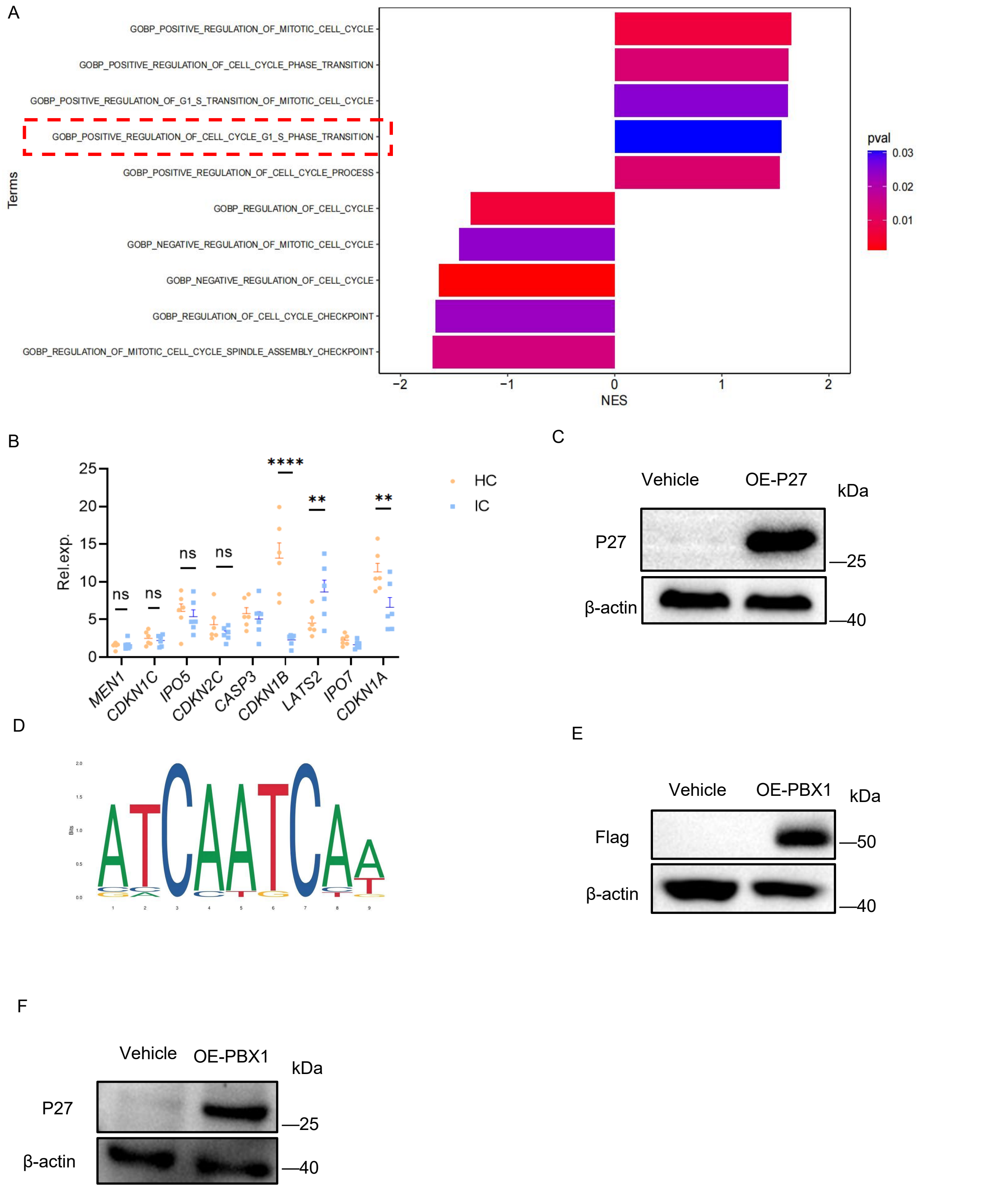

Fig.S2 Deficiency of PBX1 protein in LN mesangial cells.

(A) Top term related to cell cycle pathway enriched from differentially expressed genes in single-cell database (SYD997). (B) MCs were treated with DNA-IC isolated from plasma of SLE patients (LN MCs) and IgG isolated from healthy donors (HC MCs), mRNA expressions were detected using RT-qPCR experiment to verify the potential candidate from cell-cycle related pathway in top 20 enriched pathway from single-cell data. (C) MCs were transfected with P27 overexpression plasmid, and detected protein expression by immunoblots, β-actin was analyzed on a different gel using the same biological samples. (D) Sequence logo representing the consensus PBX1 binding motif of CDKN1B promotor obtained from JASPAR database.(E) HEK293 cells were transfected with Flag-PBX1 overexpression plasmid, and detected for PBX1 protein level by immunoblots. (F) HEK293 cells were transfected with Flag-PBX1 overexpression plasmid and detected for P27 protein level by immunoblots. *p < 0.05, **p < 0.01, ***p < 0.001, ****p < 0.0001 with ANOVA plus Turkey's method (B).

Supplementary Figure 3

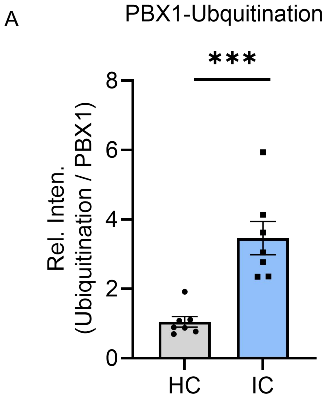

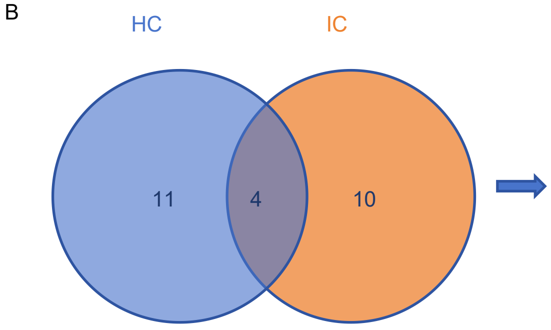

IC	Peptides
NEDD4	4
TRIM21	5
MARCH8	5
SIAH1B	6

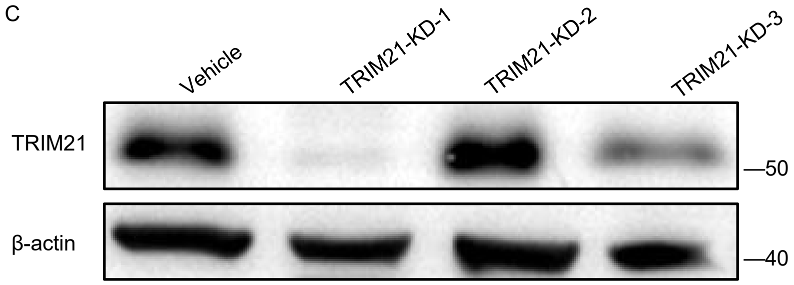

Fig.S3 Screen for candidate E3 ligase.

(A) Quantitate ubiquitination level of PBX1 protein. (B) Mass Spectrometry was conducted to screen for possible E3 ligases that bind with PBX1 in HC and LN MCs and only proteins with peptide > 1 wound be demonstrated. (C) MCs were transfected with different TRIM21shRNAs and detected for TRIM21 protein level.

Supplementary Figure 4

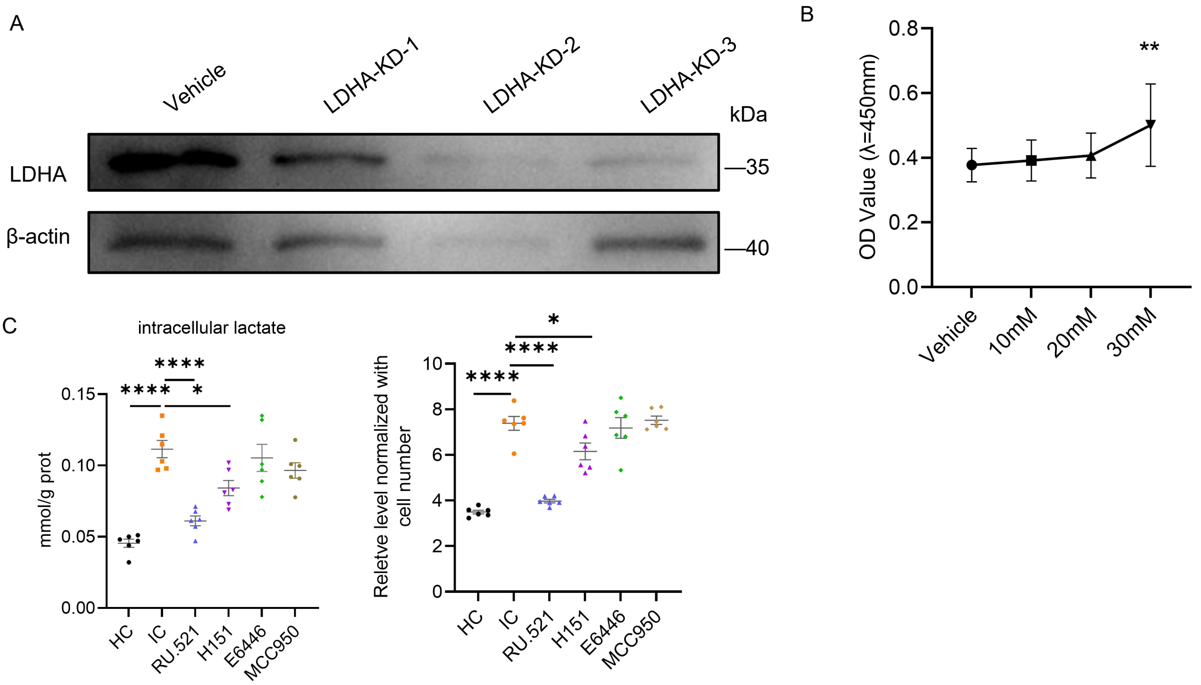

Fig.S4 Lactate production in MCs from LN patients

(A) MCs were transfected with diffierent LDHA shRNAs and detected LDHA protein level. (B) MCs were stimulated in the presence of different concentration exogenous Sodium L-lactate (10-40mM) and tested for relative cell numbers by CCK-8 kit. 10 individuals in each group. (C) Detection of lactate production levels in intracellular lactate content and extracellular lactate release in LN MCs treated with RU521 (inhibitor of cGAS), H151 (inhibitor of STING), E6446 (inhibitor of TLR7 and TLR9) and MCC950 (inhibitor of NLRP3) to explore the role of these DNA sensors in DNA-IC-induced elevated glycolysis progress in MCs (n=6, each group). lines with whiskers show the mean±SEM, *p < 0.05, **p < 0.01, ***p < 0.001, ****p < 0.0001 with ANOVA plus Turkey's method (B, C)

Supplementary Figure 5

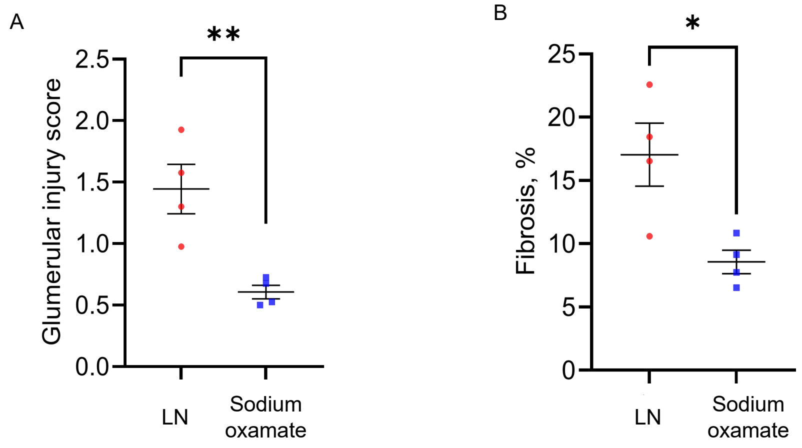

Fig.S6 Quantitation of renal pathology.

(A) The blind scoring process was executed independently by two separate pathologists. The resulting scores were then averaged, with any discrepancies requiring a third expert review. each point on the graph is an average of 20 scored glomeruli from 1 mouse (n = 4, each group). (B) A quantitative analysis of renal fibrosis was conducted using ImageJ software to calculate the percentage of total tissue area (n = 4, each group). *p < 0.05, **p < 0.01 with unpaired t-test (A, B).
